# A Cross-Country Policy-Maker Perspective on Corporate Restructuring Laws Under Stress

**DOI:** 10.1007/s40804-023-00277-3

**Published:** 2023-03-27

**Authors:** Antonia Menezes, Harry Lawless

**Affiliations:** 1 Senior Financial Sector Specialist, World Bank, Washington, D.C USA; 2 Financial Sector Specialist, World Bank, Washington, D.C USA

**Keywords:** Insolvency law, Business restructuring, COVID-era reforms, Out-of-court workouts, Corporate vulnerabilities

## Abstract

The onset of the COVID-19 pandemic saw a period of rapid experimentation with insolvency law settings, designed to prevent a wave of insolvencies. Although governments acted quickly to keep debtors out of insolvency processes, they did not alter high levels of underlying indebtedness. In this worsening economic climate characterized by low growth, high inflation, fiscal tightening and high indebtedness, it appears, in certain countries, that these measures may have deferred, rather than prevented, high insolvency levels. A key economic legacy of the COVID-19 pandemic is the extensive fiscal stimulus and the resulting budgetary constraints this has placed on governments. In this context, there is increasing evidence of the importance of frameworks for out-of-court debt workouts as a complement to formal corporate restructuring frameworks.

## Introduction

The global economic impact of the COVID-19 pandemic continues to be felt, almost 3 years on. The 3.3% global contraction in output in 2020 was the largest global recession since World War Two.^1^ It is estimated that the pandemic has cost about 4 years of progress in the pursuit of ending extreme global poverty.^2^ Indeed, 2020 was the first year in 20 years in which extreme poverty rates rose.^3^ The negative economic impact of the pandemic was also broad based, affecting more than just those in or near extreme poverty. Between 2019 and 2021, the average income of the poorest 40% fell by 2.2%, and the average income of the richest 40% fell by 0.5%.^4^ While most rich-world countries were able to return to pre-pandemic levels by the beginning of 2021, in many emerging and developing economies, recovery will take longer. Per capita income lost in 2020 is unlikely to be fully recouped by 2022 in about two thirds of emerging market and developing economies (EMDEs), including three quarters of fragile and conflict-affected low-income countries.^5^

COVID-19 and subsequent economic shocks have meant that the global economy is now facing serious economic headwinds. The World Bank’s most recent biannual ‘Global Economic Prospects’ report assessed global economic conditions, factoring in the impact of recent shocks of inflation and the invasion of Ukraine. The report forecasts that global growth is projected to slow to its third-weakest pace in nearly three decades, overshadowed only by the 2009 and 2020 global recessions.^6^ Investment growth in emerging market and developing economies is predicted to remain below its average rate of the past two decades, and any additional adverse shocks could push the global economy into recession.^7^ Information from between May and August 2022 shows high inflation in almost all low- and middle-income countries.^8^ Inflation is expected to peak in mid-2022 and then decline, but to remain elevated even after these shocks subside and monetary policies are tightened further. The share of high-income countries with high inflation has also increased sharply, with about 85.7% experiencing high food price inflation.^9^ This phenomenon of slower-than-average growth coupled with higher-than-average inflation activates a risk of stagflation.^10^ The stagflation of the 1970s required steep increases in interest rates by major advanced-economy central banks to quell inflation, which triggered a global recession and a string of financial crises in EMDEs. The existence of two further indicators points to the risk of a financial crisis in the medium term. First, every global recession since 1970 was preceded by a significant weakening of global growth in the previous year.^11^ Second, historically, global recessions have coincided with sharp economic slowdowns or recessions in several major economies.^12^

A key economic legacy of the COVID-19 pandemic is the extensive fiscal stimulus and the resulting budgetary constraints this has placed on governments. The global value of stimulus since March 2020 is estimated by the IMF at USD $13.8 trillion.^13^ This represents 10-15% of annual global GDP.^14^ In some countries as much as 30% of GDP was committed to COVID-19 stimulus.^15^ A study by the Federal Reserve suggests that the stimulus measures as a whole did contribute to inflation, because they increased demand within the economy without facilitating any consequent increase in supply to meet this additional demand.^16^

As such, a critical feature of the current macro-economic climate is record public and private debt levels. Rising global borrowing costs are heightening the risk of financial stress among the many emerging market and developing economies that over the past decade have accumulated debt at the fastest pace in more than half a century. One of the risks of a high sovereign debt burden is the corporate spillover effects, such as higher taxation, reduced subsidies and the crowding out of private lending by banks affected by sovereign risk.^17^ Compounding these risks is that private credit to non-financial corporations as a share of GDP was already at historic highs before COVID-19^18^, and now (after many firms borrowed to tackle liquidity problems in the crisis) remains above pre-pandemic levels for many countries.^19^ This puts further financial strain on corporates and increases the risk of corporate debt overhang, thereby deterring new lending and stifling investment.^20^

The picture that emerges is of a real risk of significant widespread corporate financial distress in many economies. It is therefore critical that policy-makers evaluate the efficacy of procedures provided by the state for the treatment of corporate over-indebtedness, in particular restructuring frameworks. Many countries have experimented with restructuring law reform during the pandemic period, against the backdrop of widespread and severe liquidity crises for companies across many sectors during the first year of the pandemic.^21^ Part 2 explains how governments first moved to avoid mass insolvency filings, and then pursued a combination of fiscal measures and restructuring law reforms, and identifies some key trends in relation to the latter. Part 3 considers what lessons can be learned from this pandemic experience, with a view to informing the vital task of evaluating corporate restructuring regimes. Part 4 concludes.

## The Pandemic Response and Corporate Restructuring Reforms

In conjunction with the stimulus measures, a number of other wide-ranging emergency measures were put in place during the pandemic to address possible economic risks. Governments were concerned by the possibility of mass corporate insolvencies, particularly in sectors significantly affected by lock-downs and travel restrictions, such as tourism and hospitality. Many responded by implementing changes to debt enforcement and insolvency regimes, encouraging business ‘hibernation’ and making it harder, in most cases, for creditors to initiate insolvency proceedings. The categorization and analysis of the temporary emergency measures implemented during the pandemic to stave off a wave of debt enforcement and insolvency actions has been well captured by the World Bank and partner organizations.^22^ The most common reforms are summarized in Table 1 below.^23^ Some of the early evidence of the effects of these measures in the period October 2019 to July 2020 was that insolvency filings fell (in the jurisdictions examined) in the first 6 months of 2020, contrary to expectations of a large rise.^24^

**Table 1:** Summary of COVID-era insolvency reforms

	Type of reform	Examples of different types of this reform	Frequency of reform
1	Increasing barriers for creditor-initiated insolvency filings	Raising or introducing threshold requirements for creditors to initiate insolvency proceedings; Suspending specific creditors’ rights to initiate insolvency proceedings; Increasing response timelines for debtors.	Almost half of surveyed jurisdictions
2	Suspending duty to file for insolvency	Suspension of directors’ duty to initiate insolvency; Relaxation of wrongful trading provisions.	Less than one third of surveyed jurisdictions
3	Debt repayment measures	Addressing the prospects of repayment (e.g., extending the repayment terms of loans in Portugal); Addressing the effects of non-payment (e.g., prohibiting the acceleration of contractual terms in France); Suspending judicial proceedings (e.g., Italy); Suspending the execution of certain assets owned by individuals (e.g., Bulgaria).	More than three quarters of surveyed jurisdictions

Although governments acted^25^ quickly to keep debtors out of insolvency processes, they did not alter high levels of underlying indebtedness. For example, the most common form of intervention highlighted in the table below—debt repayment measures—operated to defer rather than to fix the inability of debtors to meet their obligations. An effect of this is that debtors (who, as indicated above, may have entered the crisis with already high debt levels) may have exited the crisis with a larger debt burden than on entry. There is therefore now a heightened need for robust corporate debt restructuring systems to help deal with the ensuing corporate debt overhang and the expected rise in the number of businesses in financial distress. Consistently with this, many countries have recently implemented corporate restructuring reforms, including with the aims of (i) helping address large, complex restructurings more efficiently; (ii) encouraging restructurings at an early stage; and (iii) providing suitable frameworks to meet the needs of smaller firms.

Some reforms were conceived of during the pandemic; other countries accelerated already-planned reforms to their corporate restructuring frameworks.^26^ For example, to reduce reliance on courts constrained by the COVID-19 crisis, Colombia and Poland introduced insolvency procedures that were meant to be available for about 2 years and that limited (in Poland) or eliminated (in Colombia)^27^ the need for court involvement in the restructuring procedure. In Poland, upon the expiration of this temporary measure, amendments to the Polish insolvency and restructuring laws came into force and included, among other changes, a modified version of the restructuring procedure introduced during the pandemic.^28^ In the Netherlands, a new pre-pack procedure had been planned since mid-2019 (against the backdrop of the European Restructuring Directive), but at the beginning of the COVID-19 outbreak, it was marked as urgent by the Ministry of Justice. It was adopted, without a sunset clause, in early 2021, enabling debtors to offer their creditors and shareholders tailor-made restructuring plans outside a formal insolvency procedure and to have the plans confirmed later by the court in a formal proceeding. In Singapore, new, temporary processes were adapted from the Insolvency, Restructuring and Dissolution Act of 2018 to assist micro and small companies that required support to restructure their debts to rehabilitate the business. Recently, the Ministry of Law of Singapore extended the availability of these processes by 18 months, to end on 28 January 2024 instead of 28 July 2022.^29^ Australia^30^ and Myanmar^31^ also introduced simplified procedures that were tailored for small businesses and, unlike in Singapore, were not time-bound. These procedures for micro and small enterprises (MSEs) generally featured simplified access and plan approval mechanisms and reduced costs of engaging facilitators or insolvency practitioners.

Country experiences suggest that complementing formal, court-supervised restructuring frameworks with more informal negotiation-based tools can encourage early restructuring, when a business is more likely to be turned around. Informal workouts are often said to operate ‘in the shadow of the law’, meaning that their success is largely driven by the credible alternative of a formal insolvency proceeding, which typically is more expensive and time-consuming. In tandem with COVID-19 temporary emergency measures or upon the expiration of these measures, several countries amended their insolvency laws to provide distressed firms with variations of hybrid (meaning a combination of formal in-court and informal out-of-court processes)^32^ or preventive restructuring procedures, for instance, Colombia, Germany, the Netherlands, Poland and the United Kingdom.^33^ Simplified restructuring and liquidation processes were also put in place in several countries, targeting micro, small and medium-sized enterprises (MSMEs), which typically are more vulnerable than larger corporates, for example, in Australia, Myanmar and Singapore.

While there are important differences between the new restructuring procedures that were adopted during the pandemic, some common features can be distilled. These include:

- Private negotiation of a restructuring agreement with limited court role;
- The possibility of obtaining a court-ordered stay or a time-limited moratorium on individual enforcement actions;
- The possibility of accessing the procedure before the debtor is in a state of insolvency;
- Provision for making the agreed-upon restructuring plan binding on dissenting minority creditors, including on dissenting classes of creditors;
- The debtor remaining in control of day-to-day business operations;
- Protection for new financing from avoidance actions.^34^

For the most part, these features are consistent with international best practice, in particular the World Bank’s insolvency standard, the Principles for Effective Insolvency and Creditor/Debtor Regimes.^35^ Principle B4.3 suggests it may be appropriate in the context of a systemic crisis for informal rules and procedures to be supplemented by interim framework enhancement measures in order to address the special needs and circumstances encountered with a view to encouraging restructuring. However, such measures should be interim only and interim measures are typically designed to cover the crisis and resolution period without undermining the conventional proceedings and systems. Access to procedures in the pre-insolvency stage is countenanced by Principle C4.4, which suggests access should be available at the stage of either ‘insolvency or financial difficulty’. As for the debtor retaining control of the business, the Principles do not specifically recommend this, but note in Principle C6 that it is one of the available approaches to the management of a distressed company.^36^

The pandemic also promoted greater automation of court services. In many jurisdictions, court activity was limited or suspended during the pandemic in order to enforce social-distancing measures and contain the pandemic. Many courts were allowed to operate at restricted capacity or to handle only urgent matters. This tended to accelerate developments in court automation and digital services.^37^ For example, in Canada, the Ontario Superior Court of Justice, the country’s largest venue for insolvency proceedings, declared that all urgent matters would be conducted either in writing or by teleconference or videoconference, unless the court orders otherwise.^38^ It is not clear yet to what extent digital services were actually used during the pandemic and will continue to be used in insolvency cases post pandemic, but anecdotally, such developments appear likely to be preserved in many countries. They are generally seen as enabling more efficient, swifter proceedings and as improving access to justice for all stakeholders.

## Lessons Learned

The temporary relief measures (in combination with fiscal stimulus) appear to have been effective in forestalling a rise in corporate insolvencies and associated non-performing loans (NPLs), at least in the short term. In most jurisdictions where data is available, insolvency filings fell during the pandemic and have yet to return to pre-pandemic levels.^39^ Similarly, rates of NPLs have not yet risen as expected. Indeed, in Europe, in January 2022, they were at historic lows.^40^ However, two questions remain open: first, whether widespread corporate distress, and potentially a broader global economic crisis, has been prevented or merely postponed; and secondly, could we have avoided high insolvency and NPL levels anyway, without putting such expensive and disruptive measures in place?

On the first question, it is still unclear as to whether the measures employed during the pandemic have avoided widespread corporate distress or merely delayed it. Given the numerous predictions of a tidal wave of insolvencies in the early stages of the pandemic, which did not materialize, forecasts appear to be less concrete today. Nonetheless, the economic headwinds described above certainly provide grounds for apprehension that a rise in insolvencies is probable in the medium term. In previous financial crises, insolvencies have taken time to materialize—about thirteen quarters from the onset of the crisis.^41^ On the other hand, it is also possible that future economic turbulence will be minor, localized, regional or mirror the uneven economic recovery from the pandemic, in which rich-world countries have recovered and are set to continue recovering faster.

On the second question, about the necessity or utility of the emergency insolvency measures, some criticisms have been made regarding the unprecedented scale and cost of the measures, which in some countries were abused over the course of the pandemic.^42^ However, the longer-term effects of these measures remain to be seen, such that a cost-benefit assessment is impossible to make at this time. There is also ongoing analysis as to whether the emergency measures alone were effective in staving off the tidal wave of bankruptcies, or if this can be attributed to other socio-economic and cultural factors, including an attitude of forbearance.^43^ In countries that implemented emergency measures and where insolvency filings did not rise with the onset of the pandemic, regulators appear to attribute this phenomenon to a range of factors. In Canada, for example, financial and liquidity supports to businesses, and patience and forbearance by lenders—including a willingness and expertise to engage in informal workouts—were represented as key explanatory factors in a survey of regulators by the International Association of Insolvency Regulators.^44^

Although we cannot at this stage fully answer these ‘big picture’ questions about the policy response to pandemic-related distress, there are some lessons that we think can already be learned from the pandemic experience. First, there is increasing evidence of the importance of frameworks^45^ for out-of-court workouts as a complement to formal corporate restructuring frameworks. This was a key finding of a 2022 survey of member countries conducted by the Financial Stability Board (FSB).^46^ There is also anecdotal evidence in many regions suggesting that NPLs did not increase extensively during the pandemic because of the large amount of informal workout activity taking place amongst lenders and borrowers that did not involve a formal proceeding. The impact of these frameworks is difficult to quantify. Indeed, the FSB report notes that one of the challenges in measuring the effectiveness of such informal workouts in practice is the non-availability of data, since they typically occur confidentially.^47^ Nonetheless, many governments see the benefit in having more informal, procedurally lighter and cost-effective workout regimes that complement more formal processes, particularly if NPLs should rapidly increase. It is anticipated that out-of-court workouts and pre-insolvency negotiations will continue to play an increasingly important role. This year, the World Bank published a revised version of its Toolkit for Corporate Workouts.^48^ This emphasizes that the availability of a range of country-tailored workout frameworks is particularly important for the purpose of sorting viable from non-viable firms,^49^ a task that was made more difficult during the pandemic by governments having effectively underwritten so many businesses through fiscal stimulus. As we exit from the pandemic, the efficacy of these frameworks will depend on them being used for more than the mere postponement or rescheduling of debt obligations. Operational as well as financial restructuring may be required to avoid the persistence of ‘zombie’ firms.^50^

Secondly, governments should prioritize reform of their formal insolvency frameworks outside of crisis periods, rather than waiting for a crisis before acting. There are obvious risks in enacting emergency legislation in a short timespan in an area of policy decision-making that can have serious repercussions for society. There are clear benefits in being able to reform legislation in a considered and careful manner. In normal times, there should be emphasis on a variety of both restructuring and liquidation tools to cater to different market segments. For example, there is an increasing acknowledgement that conventional insolvency frameworks are unwieldy when applied to small businesses, and this is being reflected in specific insolvency frameworks for small businesses.^51^ It is also important to acknowledge that there is no ‘one size fits all’ insolvency framework or set of frameworks suitable for all jurisdictions: legal ‘settings’ must be adjusted to the broader legal and socio-economic environment of a particular country, and the most significant future improvements may occur by way of capacity building (investing in legal institutions) rather than ‘law on the books’ reform. By way of example, in a country where the default decision for creditors is to enforce upon security, and the insolvency system is feared or mistrusted, finer distinctions regarding out-of-court as opposed to partly ‘in-court’ workouts become less relevant than the more elementary problem of usage of the insolvency system in general.

Thirdly, the priority of government claims in the ‘waterfall’ of claims (i.e., the distributional rules provided by insolvency law) is highly relevant to restructuring outcomes, and likely increasingly so. A current example in several jurisdictions is the treatment of the obligations of a company or individual to government (whether taxation or fines, etc.) in the statutory order of priorities contained in a country’s insolvency law. The impact of tax priorities has been more significant in recent times due to generally increasing levels of taxation. Accordingly, tax claims have increasingly consumed a higher share of insolvency estates. It remains common for tax claims to rank lower than claims of secured creditors,^52^ and prior to the pandemic there was a trend towards reducing the priority of tax claims in efforts to strengthen creditors’, particularly secured creditors’, rights.^53^ However, the trend is far from absolute, and we anticipate that as the need to secure government balance sheets becomes more urgent, the inclination to uphold government priorities or Crown preferences will possibly be increasingly attractive in many EMDEs. Importantly, tax collection agencies commonly do not—whether as a matter of their enabling legislation or merely as a policy decision—restructure debts in the same way that an ordinary creditor would. It is possible that by diminishing creditors’ recovery in liquidation processes, such policy decisions could encourage creditors’ willingness to restructure (because they know they will see little or no return from a liquidation). However, on the flipside, super priority of government claims may deter creditors from entering into any insolvency process—restructuring or liquidation—on the grounds that their recovery is ultimately eroded.

Fourthly, courts matter. Corporate restructuring during the pandemic demonstrated the importance of effective court systems, particularly in developing countries. The aviation sector is of particular interest and may serve as something of a litmus test for the effectiveness of the court system in developing countries, since the financial and operational structure of airlines is complex, and airlines are typically nationally significant, including in terms of contribution to employment. An approach in some EMDEs in the early period of the pandemic was to ‘kick the can down the road’, extending court deadlines and delaying decisions on operational restructuring. This occurred in the case of Air Mauritius, with multiple court deferments in the course of 2020.^54^ The ultimate result in late 2021 was an emergence from restructuring following a substantial bailout by the government. Such cases do lend support to the proposition that rapid bailouts may have been preferable at least in some cases to costly deferral, potentially impacting stakeholder recovery.^55^ Countries need to prioritize institutional reform and ensure that their courts are efficient and robust.

Finally, the role of technology in restructurings is likely set to increase and will be particularly important for MSEs. For example, the 2019 EU Directive on Preventative Restructuring Frameworks^56^ requires European Union member states to establish early-warning tools (EWTs) that would signal to debtors their risk of financial distress early. Technology-based tools like this could give MSEs greater access to financial expertise by linking small business debtors with low-cost expertise, filling information gaps between MSEs and lenders, and facilitating restructuring negotiations.^57^ Additionally, the emergence of Online Dispute Resolution (ODR)^58^ has given new platforms for facilitating restructuring negotiations through mediation-like processes. Although evidence of the current use of ODR in this context is limited, online mediation has the potential of being particularly beneficial in cross-border situations and facilitating negotiations when multiple parties are involved. It can facilitate scheduling of mediation conferences and save participants’ travel costs, make sharing of documents easier, and, with advancement of modern technologies, such as Blockchain, AI, cloud, Big Data and other technologies, assist with the processing of complex information of multiple creditors and even modeling of alternatives.^59^

## Conclusion

The extensive fiscal measures introduced in response to the COVID-19 pandemic to protect economies appear to have forestalled immediate financial collapse, but they have also contributed to economic conditions that have exacerbated corporate vulnerabilities. Companies must now face new challenges, including those associated with the energy crisis and the war in Ukraine, which have coincided with the phasing out of many emergency measures used during the pandemic, such as payment moratoria. Corporate leverage remains high in many countries, and there is a plain risk that many corporates will prove unable to meet their debt-servicing obligations. Over the course of the pandemic, many countries tried to strengthen their corporate restructuring regimes in some key ways, including putting in place tools to make the restructuring process more efficient, and introducing specialized regimes to deal with smaller corporates. Yet there is more work to be done to ensure that the tools introduced or reformed during the crisis are fit for purpose in their particular context, and that the institutional framework necessary for their success is in place.

## Footnotes

1 World Bank (2022a).

2 World Bank (2021a).

3 Ibid.

4 World Bank (2021b).

5 World Bank (2021c).

6 World Bank (2022b).

7 Ibid.

8 World Bank (2022c).

9 Ibid.

10 World Bank (2022d).

11 World Bank (2022e).

12 Ibid.

13 OECD (2021).

14 World Bank (2022f).

15 Elgin et al. (2020).

16 Federal Reserve (2022).

17 European Central Bank (2018).

18 Feyen et al. (2020).

19 In emerging market economies, it was at about 111% of GDP in the fourth quarter of 2021, lower than a peak of about 121% in 2020, but still significantly higher than 2019 levels – about 103%. Bank for International Settlements (2022).

20 World Bank (2022g).

21 Data from the World Bank Group’s COVID-19 Business Pulse Surveys Dashboard shows the constraints that firms in EMDEs faced in their ability to service debt. According to this data, between October 2020 and March 2021 and out of the sample of 38 countries, the share of firms that had fallen or were expected to fall into arrears in any of their outstanding liabilities in the following 6 months ranged from about 40% in Europe and Central Asia to over 50% in Sub-Saharan Africa, East Asia & Pacific, Latin America & Caribbean and MENA. Among the micro and small firms of Sub-Saharan Africa and MENA, that share was higher – nearly two thirds.

22 World Bank and Insol International (2021).

23 World Bank (2022h).

24 Muro (2021).

25 All data from: Muro (2021).

26 See Menezes and Gropper (2021).

27 Law 1116 of 2006 (‘Law 1116’).

28 Allen & Overy (2022).

29 Singapore Ministry of Law website: https://www.mlaw.gov.sg/news/press-releases/final-extension-of-application-period-for-the-simplified-insolvency-programme (accessed October 2022).

30 Corporations Amendment (Corporate Insolvency Reforms) Act 2020.

31 Insolvency Law 2020.

32 It should be noted that there are no standardized definitions and terminology of the different forms that corporate workouts can take. For the purposes of this paper, the terminology conforms to that used in previous World Bank publications.

33 For a discussion regarding the different types of workouts and the spectrum of insolvency tools from informal to formal, see World Bank (2021d).

34 See Menezes and Gropper (2021).

35 World Bank (2021d).

36 The other options being exclusive control by an insolvency representative, and supervision of management by a supervisor or insolvency representative.

37 Rooney (2021).

38 Ibid.

39 Muro (2021); American Bar Association (2022).

40 Martin et al. (2022).

41 Muro (2021).

42 United Nations (2021).

43 https://www.brookings.edu/research/fed-response-to-covid19/ (accessed October 2022).

44 World Bank, INSOL International, IAIR (2022), p 99.

45 This broad term is meant to encapsulate legislative arrangements, but also quasi- or non-legislative arrangements including central bank guidance or inter-creditor agreements.

46 Financial Stability Board (2022), p 6.

47 Ibid.

48 World Bank (2021e). More recently, the World Bank assembled the Insolvency and Creditor/Debtor Regimes Task Force to consider whether amendments were necessary to modernize the Principles for Effective Insolvency and Creditor/Debtor Regimes as they pertain to corporate workouts.

49 Ibid.

50 Liu (2022).

51 For a review of such frameworks, see Gurrea-Martinez (2021).

52 Australia, Singapore, New Zealand.

53 This trend was in line with UNCITRAL’s recommendation that insolvency laws should minimize priorities that are not based on commercial bargains (UNCITRAL (2004), p 13) and that the priority of claims should be subject to clear rules and a predictable and established process, World Bank (2021d), Principle C1. See also Principles A6 and C12.

54 Air Mauritius (2021).

55 van Zwieten et al. (2021).

56 Directive (EU) 2019/1023 of the European Parliament and of the Council of 20 June 2019 on preventive restructuring frameworks, on discharge of debt and disqualifications, and on measures to increase the efficiency of procedures concerning restructuring, insolvency and discharge of debt, and amending Directive (EU) 2017/1132 (Directive on restructuring and insolvency) OJ L 172, 26.6.2019.

57 See Menezes et al. (2020).

58 ODR is described as ‘the application of information and communications technology to the prevention, management and resolution of disputes’. See Menezes et al. (2020).

59 See Atkins (2021).
